# An engineered ferritin nanocage co-delivery system for targeted hair cells protection and hearing loss attenuation

**DOI:** 10.7150/thno.131600

**Published:** 2026-07-13

**Authors:** Junying Zhang, Zhiwei Zheng, Jiaru Zhang, Xinyuan Ma, Zihao Li, Hongyan Lin, Guohui Nie, Minmin Liang, Hui Ding, Yingzi He

**Affiliations:** 1 ENT Institute and Otorhinolaryngology Department of Eye & ENT Hospital, State Key Laboratory of Medical Neurobiology and MOE Frontiers Center for Brain Science, NHC Key Laboratory of Hearing Medicine (Fudan University), Shanghai, P. R. China.; 2 Experimental Center of Advanced Materials, School of Materials Science & Engineering, Beijing Institute of Technology, Beijing, P. R. China.; 3 Department of Ophthalmology, Beijing Chaoyang Hospital, Capital Medical University, Beijing, P. R. China.; 4 School of Basic Medical Sciences, Shanghai Medical College, Fudan University, Shanghai, P. R. China.; 5 Department of Otolaryngology and Institute of Translational Medicine, Shenzhen Second People’s Hospital, the First Affiliated Hospital of Shenzhen University Health Science Center, Shenzhen, P. R. China.

**Keywords:** ferritin, drug delivery, hair cells, hearing loss, nanomedicine

## Abstract

**Rationale:**

Hearing loss is a common sensory disorder with few treatment options, mainly due to the blood–labyrinth barrier and lack of effective targeted delivery systems. Small molecules such as LLY283 and RG108 protect hair cells (HCs), but clinical use is limited by poor cellular uptake, off-target toxicity, and inefficient cochlear delivery.

**Methods:**

We developed a ferritin (Fn)-based nanocage (LR@Fn) to co-deliver RG108 and LLY283 via local injection through the round window membrane. Fn offers natural HC targeting, a hollow cavity, and good biocompatibility. Drugs were loaded using a pH-triggered strategy while maintaining structural and colloidal stability.

**Results:**

In neonatal cisplatin and adult noise-induced hearing loss models, LR@Fn reduced HCs loss, synaptic damage, and apoptosis more effectively than dexamethasone. LR@Fn treatment activated the* Wnt/β-catenin* pathway, which may contribute to the observed protective effect.

**Conclusions:**

LR@Fn is a biocompatible ferritin-based platform for targeted inner-ear therapy, showing potential for multiple forms of hearing loss.

## Introduction

Hearing loss affects more than 466 million people worldwide and is a major cause of sensory disability. According to the World Health Organization, disabling hearing loss affects nearly 5% of the global population, with approximately 90% of cases classified as sensorineural hearing loss resulting from irreversible damage to cochlear sensory hair cells (HCs) or auditory neurons 1. Current management of hearing loss relies primarily on hearing aids, pharmacological treatment, and surgical intervention. Cochlear implantation is currently the main clinical option for patients with severe-to-profound sensorineural hearing loss who do not obtain sufficient benefit from hearing aids 2. Although cochlear implants can restore auditory perception, the procedure requires surgical placement of electrode arrays into the cochlea and is accompanied by surgical risks, substantial medical resources, and prolonged rehabilitation. These limitations have driven interest in strategies that preserve or restore cochlear sensory cells and auditory neurons. Several HC-protective strategies, either alone or in combination, have shown beneficial effects in experimental models of hearing loss.

Other classes of therapeutics have also been investigated for hearing restoration. Stem-cell therapies 3, growth factors 4, and gene-based interventions 5-7 have all been evaluated as potential treatments for inner-ear disorders. Delivery to the inner ear remains a major obstacle for these therapeutic strategies. Systemically administered agents often show poor penetration into the inner ear because of the blood–labyrinth barrier 8-10. Achieving sufficient cochlear accumulation without increasing systemic exposure remains difficult. Many nanoparticle-based delivery systems rely on antibodies, peptides, or other targeting ligands to enhance uptake by cochlear HCs 11-13. Questions regarding long-term biosafety and clinical translation still exist for many synthetic nanomaterials. In addition, ligand modification increases manufacturing complexity and may complicate quality control 14-18. Ferritin (Fn) offers a different strategy for cochlear drug delivery. As an endogenous protein assembly, Fn exhibits good biocompatibility and structural stability, and it has been reported to accumulate in cochlear HCs. The hollow cavity of Fn allows encapsulation of therapeutic agents for cochlear delivery 19, 20.

Multiple approaches have been explored for hearing restoration, including hair-cell regeneration, neuronal repair, epigenetic modulation, and stem-cell-based therapies 21. Examples include *Atoh1*-based gene therapy 22, 23, modulation of *Notch* signaling 24, 25, and several emerging small-molecule 26-28 and cell-based interventions 29, 30. Despite these advances, therapeutic efficacy often varies considerably among different forms and severities of hearing loss because the underlying cellular and molecular landscapes are highly heterogeneous. Consequently, identifying broadly applicable therapeutic agents capable of protecting cochlear cells across diverse pathological conditions remains an important objective in the field.

Our previous work showed that LLY283, a PRMT5 inhibitor, attenuated cisplatin-induced cochlear injury 31 (Table S1). Treatment reduced apoptosis and mitochondrial reactive oxygen species (ROS) accumulation in cochlear cells and spiral ganglion neurons 32, 33. Although LLY283 provided significant otoprotection, its effects varied across cochlear regions and were more pronounced in the low-frequency area.

The regional difference in LLY283-mediated protection led us to investigate additional epigenetic regulators that might improve protection in the basal cochlea, which is responsible for high-frequency hearing. Among the compounds examined, RG108 attracted particular interest because it targets a distinct epigenetic pathway 34. RG108 inhibits DNA methyltransferases (DNMTs) and has been reported to reduce DNA methylation, thereby influencing genes involved in hair-cell survival and maintenance (Table S1). Increasing evidence indicates that abnormal DNA methylation contributes to cochlear injury through effects on cellular stress responses, inflammatory signaling, and oxidative damage pathways 35, 36. In our previous studies, RG108 showed a broader protective profile than LLY283, with more evident effects in high-frequency regions of the cochlea. The complementary protective patterns of the two compounds raised the possibility that their combination could provide more comprehensive cochlear protection.

Despite the reported protective effects of LLY283 and RG108 in cisplatin-induced hearing loss, their combined efficacy in noise-induced injury has not been evaluated, and the basis of their potential interaction has not been defined. To improve cochlear delivery of these epigenetic modulators, we constructed a ferritin-based co-delivery system, termed LR@Fn (Scheme 1). Fn is a naturally occurring protein nanocage with a hollow structure, high stability, and favorable biocompatibility 37-40. LLY283 and RG108 were simultaneously encapsulated into Fn through a pH-dependent loading strategy. After round window membrane (RWM) administration, LR@Fn showed efficient cochlear distribution and prolonged local retention. The therapeutic efficacy of LR@Fn was subsequently assessed in both neonatal cisplatin-induced ototoxicity models and adult noise-induced hearing loss (NIHL) models. LR@Fn preserved cochlear HCs and auditory function more effectively than dexamethasone. RNA-seq analysis and subsequent validation experiments further revealed increased *Wnt/β-catenin* signaling after treatment. These results support Fn nanocages as a practical delivery vehicle for inner-ear therapy and identify LR@Fn as a potential strategy for hearing preservation.

## 2. Results and Discussion

### 2.1 Development and *in vitro* assessment of LR@Fn

LR@Fn was generated by co-loading LLY283 and RG108 into Fn through pH-mediated nanocage disassembly and reassembly. Recombinant human ferritin was expressed in *E. coli* and purified by ion-exchange chromatography. Electrophoretic analysis showed high protein purity, with only a single major band observed in both native PAGE and SDS–PAGE gels (Figure S1A–C).

Transmission electron microscopy (TEM) images confirmed that the characteristic spherical morphology of Fn was retained after drug encapsulation (Figure 1A). Dynamic light scattering (DLS) analysis revealed a hydrodynamic diameter of 8–10 nm for native Fn, while LR@Fn showed a modest size increase to approximately 10 ± 2 nm (Figure 1B). This size increase was consistent with successful drug incorporation into the Fn cavity. Circular dichroism (CD) spectra of Fn and LR@Fn were highly similar, indicating that drug loading did not noticeably alter the secondary structure of Fn (Figure 1C). Comparable particle-size distributions were obtained after storage and freeze-drying/reconstitution, demonstrating the stability of both Fn and LR@Fn under these conditions (Figure 1D, S2A–D).

Encapsulated drug content was determined by HPLC using calibration curves established for LLY283 (275 nm) and RG108 (224 nm). The concentrations of encapsulated LLY283 and RG108 were 60.5 μM and 56.5 μM, respectively (Figure 1E, S3). Drug content remained largely unchanged during 7 days of storage (Figure 1F). Under acidic conditions (pH 5.0), rapid drug release was observed, with most of the encapsulated cargo released within 1 h (Figure 1G).

The protective effect of LR@Fn was evaluated in HEI-OC1 cells exposed to cisplatin (Cis). LR@Fn treatment increased cell viability by approximately 3.2-fold relative to the Cis group. The protective effect was greater than that observed with free LLY283, free RG108, their combination (LR), or dexamethasone (DEX) (Figure 1H, S4). Increasing concentrations of LR@Fn led to progressively higher cell viability, consistent with a dose-dependent response (Figure 1I). The protective effect varied with drug ratio, and the 1:3 (LLY283:RG108) formulation produced the highest cell viability. Further increasing the proportion of either drug resulted in reduced protection (Figure S5). Similar results were obtained from live/dead staining experiments. Consistent with the viability data, LR@Fn treatment markedly reduced the number of dead cells after Cis challenge (Figure S6).

### 2.2 *In vitro* and *in vivo* approaches to target LR@Fn

Having established the structural stability and cytoprotective efficacy of LR@Fn, we next examined whether ferritin-mediated delivery could facilitate efficient targeting of auditory HCs. Cellular uptake was first evaluated in HEI-OC1 cells using confocal laser scanning microscopy (CLSM) (Figure 2A). HEI-OC1 cells were incubated with Cy5-labeled LR@Fn (LR@Fn-Cy5SE) for 2–20 h, followed by fluorescence imaging at the indicated time points (Figure 2B). Intracellular Cy5 fluorescence increased progressively over time, indicating continuous uptake of LR@Fn by HEI-OC1 cells (Figure 2C).

The cochlear distribution of LR@Fn was further examined in C57BL/6J mice following RWM administration. Cochleae were collected 12 h after injection and subjected to whole-mount fluorescence imaging. Strong Cy5 signals were detected in all cochlear turns, including basal, middle, and apical regions (Figure 2D). Fluorescence signals were distributed throughout the sensory epithelium. Higher fluorescence intensity was observed in both inner hair cells (IHCs) and outer hair cells (OHCs), suggesting preferential accumulation of LR@Fn in sensory HCs. Quantification of fluorescence intensity supported substantial uptake of LR@Fn by cochlear HCs *in vivo* (Figure 2E). The observed distribution pattern supports the use of Fn nanocages for cochlear drug delivery.

### 2.3 Protective effect of LR@Fn in a cisplatin-induced cochlear injury model

Cisplatin-induced ototoxicity was assessed using cochlear explants isolated from postnatal day 2 mice. Explants were pretreated with Fn, DEX, LR, or LR@Fn (100 μM), followed by exposure to 20 μM Cis for 48 h. HCs were visualized by myosin 7a staining (Figure 3A). Control explants showed a normal organ of Corti structure, consisting of one row of IHCs and three to four rows of OHCs (Figure 3B). Cis exposure caused extensive hair cell loss across the cochlear epithelium, with IHCs showing greater sensitivity than OHCs. Fn treatment alone did not noticeably reduce cisplatin-induced damage and produced results similar to those of the Cis group. DEX preserved a portion of basal OHCs but did not prevent widespread HC loss. Both LR and LR@Fn reduced hair cell loss, with LR@Fn retaining the greatest number of surviving HCs (Figure 3B, C). The overall HCs arrangement in the LR@Fn group remained close to that of untreated explants. No obvious morphological abnormalities were observed in explants treated with LR@Fn alone (100 μM). This concentration was therefore used in subsequent experiments.

Mitochondrial ROS production was assessed using MitoSOX staining. Cisplatin induced a marked increase in ROS levels within HCs. LR@Fn pretreatment significantly reduced ROS accumulation compared with cisplatin alone (Figure 3D, E; S7A, B).

Apoptosis was evaluated by cleaved caspase-3 (CC3) immunostaining. Cis exposure resulted in a pronounced increase in CC3-positive HCs throughout the cochlea. LR@Fn treatment significantly reduced apoptotic cell numbers. Similar reductions were observed in both basal and apical regions (Figure 3F, G, S7C, D). These findings indicate that LR@Fn protects cochlear HCs from cisplatin-induced injury and is accompanied by reduced oxidative stress and apoptosis.

### 2.4 LR@Fn protects against noise-induced hearing loss *in vivo*

The biosafety of LR@Fn was evaluated before *in vivo* efficacy studies. Hematoxylin and eosin (H&E) staining of major organs revealed no observable histopathological alterations in LR@Fn-treated mice compared with PBS controls. Serum biochemical indices also remained within physiological ranges, indicating the absence of detectable systemic toxicity (Figure S8A, B).

Adult C57BL/6J mice were exposed to broadband noise (2–20 kHz, 115 dB, 2 h) to induce NIHL. LR@Fn or the corresponding control formulations were injected through the RWM before noise exposure. Hearing function was evaluated by ABR testing on days 4 and 7 after noise exposure (Figure 4A). Mice exposed to noise alone showed persistently elevated ABR thresholds (~90 dB) at day 7. Only modest threshold improvement was observed in the Fn, DEX, and LR groups, mainly at 8 and 16 kHz. Among all treatment groups, LR@Fn showed the strongest recovery of ABR thresholds across the tested frequency range (Figure 4B).

Cochlear whole-mount staining for myosin 7a revealed extensive OHCs loss after noise exposure, especially in the basal and middle turns. Partial preservation was observed in Fn-, DEX-, and LR-treated groups, whereas LR@Fn maintained a higher number of OHCs across all cochlear regions (Figure S9A). IHCs loss was mainly confined to the basal turn.

Synaptic integrity was assessed by CtBP2 immunostaining. Control cochleae contained approximately 14–15 ribbon puncta per IHC. Noise exposure reduced this to 7–9 puncta per IHC. LR@Fn treatment preserved synaptic counts close to baseline levels (Figure 4C, D). Cochlear innervation was evaluated by NF and myosin 7a co-staining. Noise exposure caused pronounced loss of afferent fibers. Fn, DEX, and LR provided limited preservation, whereas LR@Fn maintained a higher density of NF-positive projections toward OHCs (Figure 4E, F). Caspase-3/7 staining showed strong apoptotic activation in the noise group. LR@Fn reduced apoptotic signals in cochlear HCs, which were better preserved than with LR alone (Figure 4G, H). Together, these results show that LR@Fn alleviated noise-induced hearing loss and was associated with improved hair-cell survival, preservation of ribbon synapses and neural fibers, and reduced apoptosis.

### 2.5 LR@Fn protects cisplatin-treated HCs via activation of the *Wnt/β-catenin* pathway

RNA sequencing was conducted in HEI-OC1 cells following Cis exposure with or without LR@Fn pretreatment. Cis treatment altered the expression of a large number of genes, which were partially reversed by LR@Fn pretreatment. Compared with Cis alone, 2,676 genes were upregulated and 1,848 genes were downregulated following LR@Fn treatment (Figure 5A), including *Wnt*-associated genes such as *Tcf7l2* and *Axin2*.

KEGG enrichment analysis of differentially expressed genes (P < 0.05, fold change ≥ 2) identified the *Wnt/β-catenin* pathway as the most significantly affected signaling axis (Figure 5B). This pathway is essential for inner ear development and hair cell differentiation during cochlear formation 41. Canonical *Wnt* signaling involves *β-cateni*n nuclear translocation and transcriptional regulation via Tcf/Lef complexes 42-45. *β-catenin* is required for hair cell development, and its loss impairs sensory progenitor differentiation 46. However, its role in cisplatin-induced hair cell protection remains unclear.

Heatmap analysis showed increased expression of *Axin2*, *β-catenin*, and *Tcf7l2* in the LR@Fn + Cis group compared with Cis alone (Figure 5C). Reverse transcription quantitative PCR (RT-qPCR) validation showed that Cis significantly suppressed the mRNA levels of these genes, whereas LR@Fn pretreatment restored their expression (Figure 5D; primer sequences in Table S2). Similar changes were observed at the protein level, Western blot analysis demonstrated that protein levels of *Axin2*, *β-catenin*, and *Tcf7l2* were reduced by Cis and recovered following LR@Fn treatment (Figure 5E).

To evaluate the functional relevance of *β-catenin* activation, the pathway was inhibited using NVP–XAV939. Blocking *β-catenin* partially attenuated the anti-apoptotic effect of LR@Fn in cisplatin-treated HEI-OC1 cells (Figure 5F), suggesting that activation of this pathway is involved in the protective effect of LR@Fn. Together, these findings suggest that activation of *Wnt/β-catenin* signaling may contribute to the protective effect of LR@Fn against cisplatin-induced hair-cell injury.

## 3. Conclusion

Previous studies have shown that RG108 and LLY283 can protect HCs and spiral ganglion neurons from cisplatin-induced injury through multiple mechanisms. However, efficient drug delivery to the inner ear remains limited by the blood–labyrinth barrier, which restricts systemic access to cochlear tissues.

In this study, Fn nanocages were engineered as a co-delivery platform for RG108 and LLY283, generating LR@Fn. Local administration via round window membrane injection enabled effective cochlear delivery in a mouse model of noise-induced hearing loss, resulting in significant therapeutic benefits without observable systemic toxicity. This strategy bypasses the inner ear barrier and improves local drug availability.

RNA-seq, RT-qPCR, and Western blot analyses consistently showed increased *Wnt/β-catenin* signaling after LR@Fn treatment, indicating a possible role of this pathway in cochlear protection. Single-drug Fn formulations (L@Fn and R@Fn) were not evaluated in this work. Future studies should determine whether co-loading provides advantages beyond those achieved by each drug individually.

LR@Fn combines the biocompatibility of Fn with efficient cochlear delivery of therapeutic agents. The loading strategy described here may also be applicable to other therapeutic molecules for cochlear diseases.

## 4. Experimental Section

### Heavy-chain Fn expression and purification

The gene encoding heavy-chain ferritin (Fn) was inserted into the pET-30a (+) vector for recombinant expression. Competent *E. coli* received the plasmid through heat shock, and transformants were picked on antibiotic-laden agar plates. The cultures were grown at 37 °C and induced with 0.5 mM IPTG upon reaching the exponential growth phase. Cells were collected after 16–18 h of induction and disrupted to release intracellular proteins. Fn was isolated from the lysate by sequential ion-exchange, affinity, and size-exclusion chromatography. Protein homogeneity was evaluated from the size-exclusion chromatography profile obtained on an AKTA system.

Protein samples were combined with loading buffer, heated at 100 °C for 10–15 minutes, and then separated on precast gels alongside molecular weight markers for SDS–PAGE. Following electrophoresis (120V), the gels were stained with Coomassie Brilliant Blue and washed. Protein purity was evaluated from the electrophoretic profiles.

### Preparation of LR@Fn nanodrugs

Take 10 mg of purified Fn and adjust the pH to 2.2 using 0.5 M HCl to dissociate the ferritin nanocages. Add LLY283 and RG108 dropwise to the solution to achieve a final concentration of 100 μM for each compound. The mixture was stirred at room temperature for two hours, then the pH was slowly adjusted back to 7.5 using 0.5 M NaOH, causing the ferritin nanocages to reassemble and encapsulate the drug molecules. The suspension was centrifuged to remove insoluble aggregates, and the supernatant was collected. The resulting LR@Fn nanocages were recovered and stored for subsequent experiments.

### TEM

Fn and LR@Fn suspensions were diluted to 1 mg/mL in PBS. A droplet of each sample was placed on a carbon-coated copper grid. Uranyl acetate was used to negatively stain the samples for 1–2 minutes before they were air-dried. Nanocage morphology before and after drug loading was observed by TEM.

### DLS

Particle size and size distribution were determined by DLS. Diluted samples were loaded into quartz cuvettes for measurement.

### CD

The samples were prepared in a 10 mM phosphate buffer with a pH of 7.4, maintaining a protein concentration of 0.2 mg/mL. Spectra were captured between 190–250 nm. After baseline correction, secondary-structure contents were estimated using DichroWeb.

### Efficiency and capacity for drug loading

Drug loading was quantified by HPLC. LR@Fn nanocages were digested with proteinase K to release encapsulated LLY283 and RG108. Drug concentrations were determined from standard calibration curves, and loading efficiency was calculated accordingly.

### Stability studies

Fn and LR@Fn were dispersed in PBS (pH 7.4) and stored under physiological conditions. Particle size was monitored by DLS after 7 days of storage. For drug-retention studies, LR@Fn samples were collected at designated time points. Drug content was quantified by HPLC using the same protocol described above, and the remaining drug percentage was calculated.

### Drug release

Drug release was examined in PBS buffer, using pH 7.4 to mimic the extracellular environment and pH 5.0 for lysosomes. LR@Fn was sealed inside dialysis bags and then left to incubate in the release medium, with gentle stirring. The release medium was sampled at set time points. Released drug content was quantified by HPLC, and cumulative release percentages were calculated.

### HEI-OC1 cell culture

HEI-OC1 cells were sustained in a high-glucose DMEM medium with 10% fetal bovine serum at 33 °C in a humidified atmosphere with 5% CO_2_.

### Cell viability assay

Assay cell viability by measuring absorbance at 450 nm using the Cell Counting Kit-8 (CCK-8). HEI-OC1 cells were seeded into 96-well plates at a density of 1 × 10^4^ cells per well and allowed to grow overnight. Cells were treated with Cis (20 μM), DEX, RG108, LLY283, LR, or LR@Fn at the indicated concentrations (0–100 μM). Test compounds were added 2 h prior to Cis treatment. After 22 hours, add the CCK-8 reagent and continue incubating for 2 hours.

Similarly, to determine the effect of *Wnt/β-catenin* inhibition, HEI-OC1 cells were pretreated with NVP–XAV939 and LR@Fn for 2 hours prior to exposure to cisplatin.

### Cellular uptake analysis

Cells of the HEI-OC1 line were seeded onto confocal dishes. Cells were incubated with Cy5SE-labeled LR@Fn for the indicated periods. Cells underwent washing with cold PBS, fixation with 4% paraformaldehyde, and counterstaining with DAPI. A confocal laser scanning microscope (CLSM) was used to study the intracellular distribution of LR@Fn.

### *In vivo* HC uptake analysis

Cochlear uptake of LR@Fn was evaluated using a round window membrane (RWM) delivery model. LR@Fn-Cy5SE (2 μL) was administered to the RWM through a postauricular surgical approach. At 12 h post-injection, administer anesthesia to the mice and euthanize them, then remove the cochleae. Immerse the cochleae in 4% paraformaldehyde and decalcify them in a 4% ethylenediaminetetraacetic acid (EDTA) solution at 4°C for 24–48 hours. Dissect the cochleae, label the hair cells, and observe them under a CLSM.

### Neonatal mouse cochlear basilar membrane culture

C57BL/6 mouse cochleae were isolated on the second day after birth (P2) and transferred to PBS. Basilar membranes were carefully dissected under a stereomicroscope to preserve the sensory epithelium. Attach the tissue blocks to Cell-Tak-coated cover slips, place them in a 4-well plate, and grow in DMEM/F12 medium with N2/B27 and antioxidants. Culture conditions are 37°C and 5% CO_2_.

### Immunofluorescence staining of the neonatal cochlear basilar membrane

Tissues were embedded in 4% paraformaldehyde, rinsed with PBS, and treated with 10% serum and 1% Tween 20 in PBS for 2 h for permeabilization and blocking. The primary antibody was incubated overnight at 4°C, and the secondary antibody was exposed for 2 h with the sample at room temperature. Stain with DAPI. Mount the samples with Fluoromount-G and image using a Leica TCS SP8 CLSM.

Primary antibodies: anti-parvalbumin (Abcam, ab32895), anti-CC3 (Abcam, ab2302), MitoSOX (Thermo Fisher Scientific, M36008), anti-myosin 7a (Proteus Biosciences, 25–6790). Secondary antibodies: Alexa Fluor 488-, 594-, and 647-conjugated antibodies against rabbit, mouse, and goat IgG (Invitrogen).

### HCs quantification

IHCs and OHCs were counted along 100 μm segments of the cochlear basilar membrane. The average number of cells was determined by selecting six segments at random from each cochlear region. Hair cell damage was defined as loss of cells or disruption of normal arrangement. Apoptotic cells were identified by Caspase-3/7 staining and quantified in the indicated cochlear regions. Oxidative stress levels were evaluated based on MitoSOX Red fluorescence intensity.

### NIHL model

The C57BL/6 mice were randomly split into six groups for the experiment: Control, Noise, DEX-Noise, Fn-Noise, LR-Noise, and LR@Fn-Noise. A postauricular surgical approach was used to deliver different formulations through the round window membrane. Two days after drug administration, mice were exposed to broadband noise (2–20 kHz, 115 dB SPL) for 2 h. Mice were maintained under standard conditions until the scheduled analyses.

### ABR measurement

Mice received an intraperitoneal injection of pentobarbital sodium (50 mg/kg) for anesthesia and were then positioned in a chamber designed to minimize noise. Recording electrodes were placed subdermally at the vertex and beneath the pinnae according to standard ABR procedures. Acoustic stimuli were generated and recorded using the TDT System III platform with SigGen/Biosig software. Stimuli at frequencies of 4, 8, 16, 24, and 32 kHz were presented as tone bursts, and the responses were averaged over 1024.

### Basilar membrane dissection of adult mouse cochlea

The mice were euthanized prior to tissue collection. A small incision was made at the apex of the cochlea to facilitate fixation, and the tissue was decalcified in EDTA solution for 48 hours. Basilar membranes were carefully dissected under a stereomicroscope and separated into apical, middle, and basal regions for subsequent analyses.

### Immunofluorescence staining of adult cochlear basilar membrane

As in the previous steps, isolate the cochlear tissue, seal the sample, incubate with the primary antibody, incubate with the secondary antibody, stain with DAPI, mount the slide, and image using a CLSM.

Primary antibodies included anti-CtBP2 (BD Biosciences, 612044), anti-neurofilament (NF; Abcam, ab72996), and anti-Caspase-3/7 (Thermo Fisher Scientific, R37111). Alexa Fluor 488-, 594-, and 647-conjugated secondary antibodies (Invitrogen) were used for fluorescence detection.

### Quantitative analysis

Hair-cell survival, MitoSOX-positive regions, and Caspase-3/7-positive cells were quantified using the same criteria applied to neonatal cochlear explants. Six randomly selected 100 μm regions from each cochlea were analyzed and averaged. Ribbon synapses were quantified by counting CtBP2-positive puncta per IHCs. Nerve-fiber density was evaluated based on NF-positive projections associated with HCs.

### Safety evaluation

Healthy C57BL/6 mice received either PBS or LR@Fn through RWM injection. Three days later, blood samples were collected for serum biochemical analysis. Major organs were harvested, fixed, and subjected to H&E staining to assess potential histopathological abnormalities.

### Animal ethics

All experiments involving animals were carried out following institutional guidelines for their care and use, with approval from the Institutional Animal Care and Use Committee (BIT-EC-SCXK(Yu)2020-0005-195).

### Western blotting

The supernatant from lysed HEI-OC1 cells was collected, and the proteins were transferred to a PVDF membrane via gel electrophoresis. The PVDF membrane was blocked and incubated with anti-Axin2 (1:1000, ab32197), β-catenin (1:4000, ab16051), and Tcf7l2 (1:1000, ab76151) (Abcam). Incubate with secondary antibodies and visualize protein bands. GAPDH (1:1000, Invitrogen, MA5-15738) served as the loading control.

### RNA sequencing and reverse transcription quantitative PCR (RT-qPCR)

The extraction and quantification of total RNA from HEI-OC1 cells were performed using an Agilent 2100 Bioanalyzer. STAR was used to align clean reads to the mouse reference genome (mm10). Genes with differential expression were pinpointed using DESeq2 based on these criteria: P < 0.05, fold change ≥ 2, and FDR < 0.01. Enrichment analyses for GO and KEGG were conducted using R packages.

To conduct RT-qPCR, total RNA was extracted with TRIzol reagent and then converted into cDNA. Quantitative PCR was carried out using SYBR Green Master Mix on a CFX96 system. The calculation of relative gene expression employed the 2^-ΔΔCt method, with *β-actin* as the reference. Primer sequences are detailed in Table S2.

### Statistical analysis

The data are shown as the mean plus or minus the standard deviation (SD). Statistical tests were conducted with GraphPad Prism 9.0. Group differences were assessed using one-way ANOVA, and a P value of less than 0.05 was deemed statistically significant.

## Supplementary Material

Supplementary figures.

## Figures and Tables

**Scheme 1:**
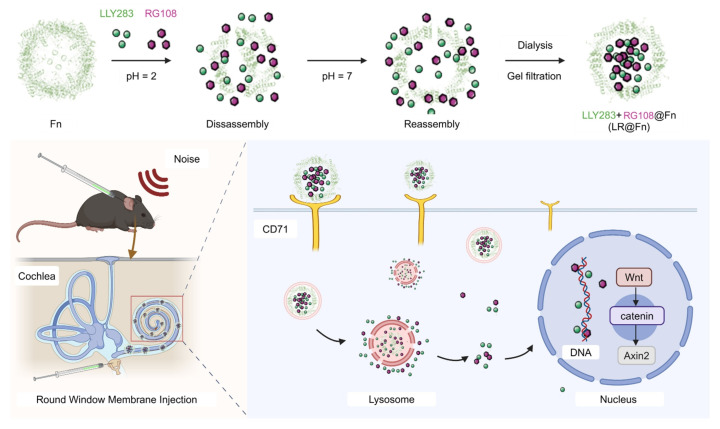
** Summary of the multifunctional targeted nanocage (LR@Fn) delivery platform.** Ferritin nanocages were disassembled under acidic conditions to co-load LLY283 and RG108, followed by pH-triggered reassembly to form LR@Fn. After round window membrane injection, LR@Fn targeted cochlear hair cells (HCs) through CD71-mediated uptake and enabled sustained intracellular drug release, thereby activating *Wnt/β-catenin* signaling to protect against cisplatin- and noise-induced hearing loss.

**Figure 1:**
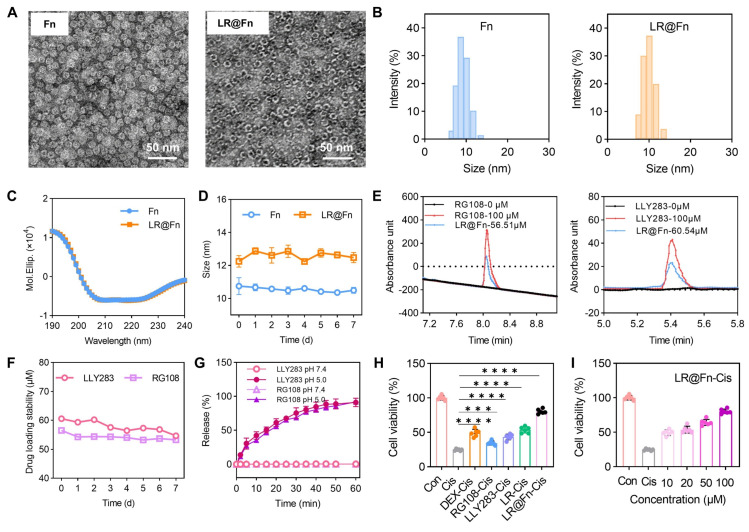
** Preparation and characterization of LR@Fn nanodrugs.** (A) TEM images of uranyl acetate–stained Fn and LR@Fn. (B) Hydrodynamic size distribution measured by DLS. (C) Circular dichroism spectra of Fn and LR@Fn. (D) Particle size stability over time. (E) Drug loading capacity of LR@Fn measured by HPLC. (F) Drug retention stability in PBS. (G) *In vitro* release profile of LR from LR@Fn under different pH conditions. (H) Viability of HEI-OC1 cells after treatment with PBS, Cisplatin (Cis), DEX, RG108, LLY283, LR, or LR@Fn. (I) Dose-dependent cytoprotective effect of LR@Fn against cisplatin-induced cell death. Data in (D), (G), (H), and (I) are mean ± SD (n=3 for D and G; n=6 for H and I) and analyzed by one-way ANOVA. Consistent results were obtained from experiments in (A–C), (E), and (F), each independently repeated three times. ***p < 0.001, ****p < 0.0001.

**Figure 2:**
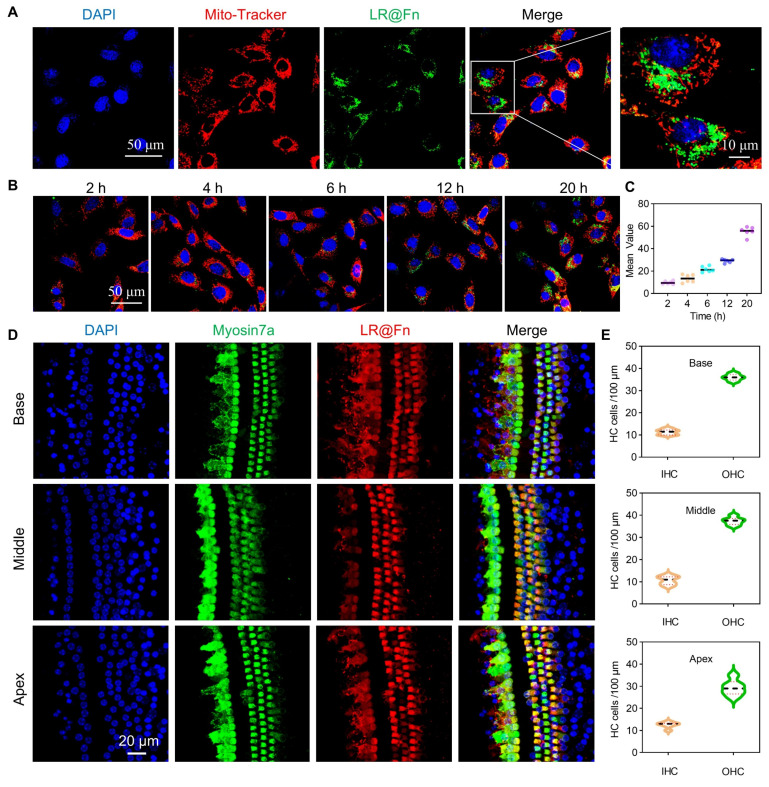
** Targeting evaluation of LR@Fn.** (A) CLSM images of HEI-OC1 cells incubated with LR@Fn for 20 h; mitochondria (red), LR@Fn (green), nuclei (DAPI, blue). (B) Time-dependent uptake of LR@Fn in HEI-OC1 cells (2, 4, 6, 12, 20 h). (C) Quantitative analysis of LR@Fn cellular uptake over time (n = 6). (D) CLSM images of LR@Fn distribution (red) in cochlear HCs across apical, middle, and basal regions; HCs labeled by myosin 7a (green), nuclei by DAPI (blue). (E) Quantification of LR@Fn-targeted HCs (n = 6). Data in (C) and (E) are mean ± SD; imaging trials were independently conducted in three repeats with consistent outcomes.

**Figure 3:**
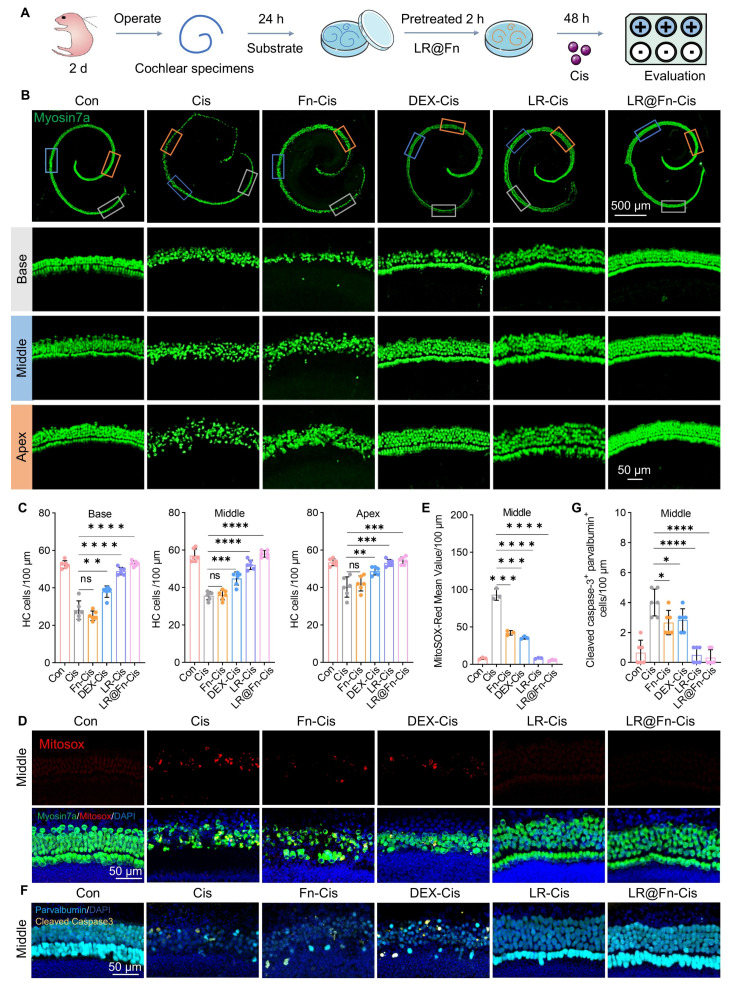
** Protective effects of LR@Fn against cisplatin-triggered hair cell damage *ex vivo*.** (A) Diagram of the culture and treatment protocol for neonatal mouse cochlear explants. (B) Cochlear HCs (myosin 7a, green) in the apical, middle, and basal regions were imaged using CLSM after treatment with control medium (Con), Cis, Fn, DEX, LR, or LR@Fn. (C) Quantification of HC survival across cochlear regions (n = 6). (D) MitoSOX staining showing mitochondrial superoxide (red); HCs labeled by myosin 7a (green), nuclei by DAPI (blue). (E) Quantification of mitochondrial superoxide levels (n = 3). (F) CLSM images of cleaved caspase-3 (CC3, yellow) in HCs. (G) Quantification of CC3-positive HCs (n = 6). Data are mean ± SD; one-way ANOVA: ns, not significant; *p < 0.05, **p < 0.01, ***p < 0.001, ****p < 0.0001.

**Figure 4:**
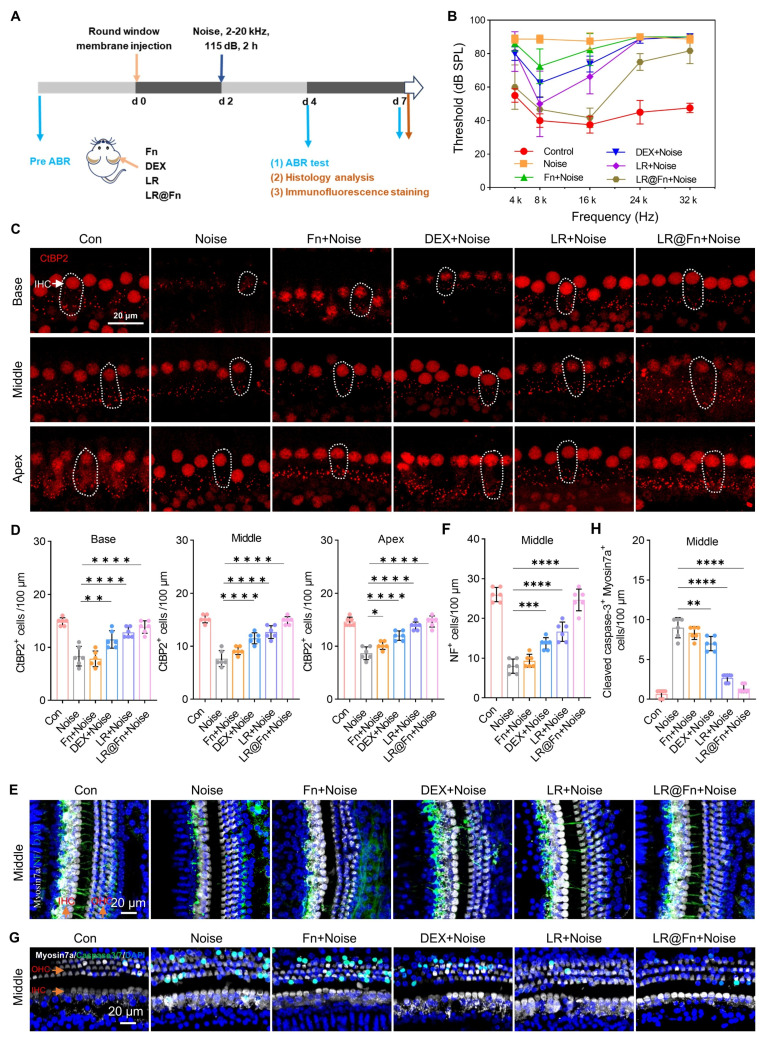
** Protective effects of LR@Fn on NIHL *in vivo*.** (A) Schematic of the NIHL model and treatment design. (B) Auditory brainstem response (ABR) thresholds at different frequencies on day 7 post-noise exposure (n = 6). (C) Confocal images of CtBP2-labeled ribbon synapses (red) in cochlear HCs. (D) Quantification of CtBP2-positive ribbon synapses in apical, middle, and basal cochlear regions (n = 6). (E) Confocal images of neurofilaments (NF, green) in cochlear HCs; HCs labeled by myosin 7a (white), nuclei by DAPI (blue). (F) Quantification of neurofilament-positive fibers in the middle cochlear region (n = 6). (G) Confocal images of apoptotic markers (green) in cochlear HCs; HCs labeled by myosin 7a (white), nuclei by DAPI (blue). (H) Quantification of apoptotic HCs (n = 6). Data are mean ± SD; one-way ANOVA: *p < 0.05, **p < 0.01, ***p < 0.001, ****p < 0.0001.

**Figure 5:**
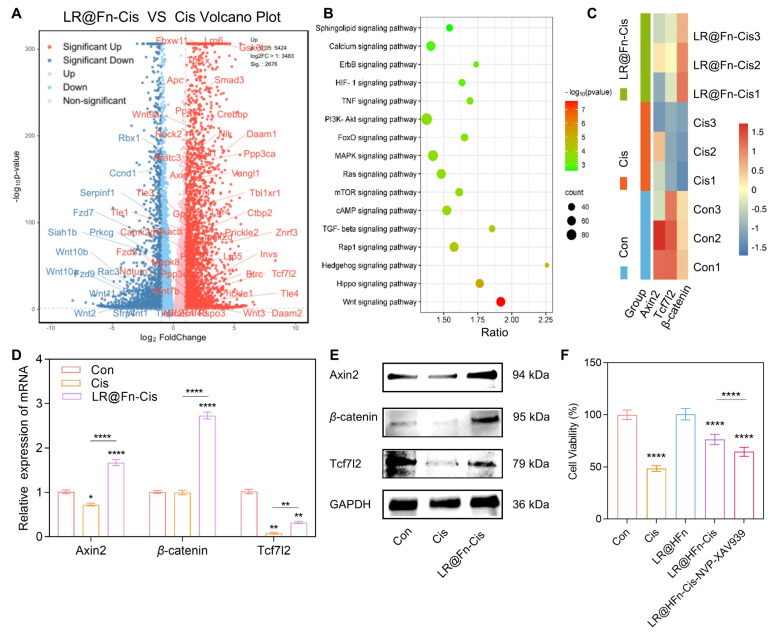
** RNA-seq analysis shows activation of the *Wnt/β-catenin* pathway in LR@Fn-mediated protection.** (A) Volcano plot of DEGs between Cis-treated and LR@Fn + Cis-treated HEI-OC1 cells. (B) KEGG analysis of DEGs. (C) Heatmap of *Wnt/β-catenin* pathway–related genes (red, over-expression; blue, down-expression). (D) RT-qPCR of *Axin2*, *β-catenin*, and *Tcf7l2* mRNA levels (n = 4). (E) Western blot analysis of *Axin2*, *β-catenin*, and *Tcf7l2* protein expression (GAPDH control). (F) Cell viability of HEI-OC1 cells subjected to NVP-XAV939, LR@Fn, and Cis (n = 4). Data are mean ± SD; one-way ANOVA. *p < 0.05, **p < 0.01, ****p < 0.0001.

## References

[1] Nieman CL, Oh ES (2020). Hearing Loss. Ann Intern Med.

[2] Geleoc GS, Holt JR (2014). Sound strategies for hearing restoration. Science.

[3] Chen W, Jongkamonwiwat N, Abbas L, Eshtan SJ, Johnson SL, Kuhn S (2012). Restoration of auditory evoked responses by human ES-cell-derived otic progenitors. Nature.

[4] Okano T, Nakagawa T, Kita T, Endo T, Ito J (2006). Cell-gene delivery of brain-derived neurotrophic factor to the mouse inner ear. Mol Ther.

[5] Landegger LD, Pan B, Askew C, Wassmer SJ, Gluck SD, Galvin A (2017). A synthetic AAV vector enables safe and efficient gene transfer to the mammalian inner ear. Nat Biotechnol.

[6] Zou B, Mittal R, Grati M, Lu Z, Shu Y, Tao Y (2015). The application of genome editing in studying hearing loss. Hear Res.

[7] Zuris JA, Thompson DB, Shu Y, Guilinger JP, Bessen JL, Hu JH (2015). Cationic lipid-mediated delivery of proteins enables efficient protein-based genome editing in vitro and in vivo. Nat Biotechnol.

[8] Nyberg S, Abbott NJ, Shi X, Steyger PS, Dabdoub A (2019). Delivery of therapeutics to the inner ear: The challenge of the blood-labyrinth barrier. Sci Transl Med.

[9] Pang X, Zhang X, Xu D, Bu C, Qiu Y, Feng P (2026). Bioactive hydrogels for the reconstruction of sensorineural hearing loss. Bioact Mater.

[10] Cheng H, Zhang B, Jiang P, Liao M, Gao X, Xu D (2025). Biomaterial-based drug delivery systems in the treatment of inner ear disorders. J Nanobiotechnology.

[11] Roy S, Johnston AH, Newman TA, Glueckert R, Dudas J, Bitsche M (2010). Cell-specific targeting in the mouse inner ear using nanoparticles conjugated with a neurotrophin-derived peptide ligand: Potential tool for drug delivery. Int J Pharm.

[12] Li L, Chao T, Brant J, O'Malley B, Tsourkas A, Li D (2017). Advances in nano-based inner ear delivery systems for the treatment of sensorineural hearing loss. Adv Drug Deliv Rev.

[13] Liao M, Zhu Q, Cheng H, Qiu Y, Xu S, Bu C (2026). Magnetic Mesoporous Nanoparticles Loaded with Lycium barbarum Glycopeptide for Targeted Therapy of Noise-Triggered Auditory Dysfunction. Adv Mater.

[14] Dai J, Long W, Liang Z, Wen L, Yang F, Chen G (2018). A novel vehicle for local protein delivery to the inner ear: injectable and biodegradable thermosensitive hydrogel loaded with PLGA nanoparticles. Drug Dev Ind Pharm.

[15] Kayyali MN, Ramsey AJ, Higbee-Dempsey EM, Yan L, O'Malley BW Jr, Tsourkas A (2018). The Development of a Nano-based Approach to Alleviate Cisplatin-Induced Ototoxicity. J Assoc Res Otolaryngol.

[16] Gu J, Chen Y, Tong L, Wang X, Yu D, Wu H (2020). Astaxanthin-loaded polymer-lipid hybrid nanoparticles (ATX-LPN): assessment of potential otoprotective effects. J Nanobiotechnology.

[17] Gu J, Wang X, Chen Y, Xu K, Yu D, Wu H (2022). An enhanced antioxidant strategy of astaxanthin encapsulated in ROS-responsive nanoparticles for combating cisplatin-induced ototoxicity. J Nanobiotechnology.

[18] Chen H, Zhang H, Dai Y, Zhu H, Hong G, Zhu C (2023). Magnetic Hydrogel Microrobots Delivery System for Deafness Prevention. Adv Funct Mater.

[19] Fan K, Cao C, Pan Y, Lu D, Yang D, Feng J (2012). Magnetoferritin nanoparticles for targeting and visualizing tumour tissues. Nat Nanotechnol.

[20] Levi S, Yewdall SJ, Harrison PM, Santambrogio P, Cozzi A, Rovida E (1992). Evidence of H- and L-chains have co-operative roles in the iron-uptake mechanism of human ferritin. Biochem J.

[21] Wang X, Xu L, Meng Y, Chen F, Zhuang J, Wang M (2024). FOXO1-NCOA4 Axis Contributes to Cisplatin-Induced Cochlea Spiral Ganglion Neuron Ferroptosis via Ferritinophagy. Adv Sci (Weinh).

[22] Gubbels SP, Woessner DW, Mitchell JC, Ricci AJ, Brigande JV (2008). Functional auditory hair cells produced in the mammalian cochlea by in utero gene transfer. Nature.

[23] Izumikawa M, Minoda R, Kawamoto K, Abrashkin KA, Swiderski DL, Dolan DF (2005). Auditory hair cell replacement and hearing improvement by Atoh1 gene therapy in deaf mammals. Nat Med.

[24] Ma J, Xia M, Guo J, Li W, Sun S, Chen B (2024). MEK/ERK signaling drives the transdifferentiation of supporting cells into functional hair cells by modulating the Notch pathway. Stem Cells Transl Med.

[25] Li W, Wu J, Yang J, Sun S, Chai R, Chen ZY (2015). Notch inhibition induces mitotically generated hair cells in mammalian cochleae via activating the Wnt pathway. Proc Natl Acad Sci U S A.

[26] Quan YZ, Wei W, Ergin V, Rameshbabu AP, Huang M, Tian C (2023). Reprogramming by drug-like molecules leads to regeneration of cochlear hair cell-like cells in adult mice. Proc Natl Acad Sci U S A.

[27] Yamahara K, Yamamoto N, Nakagawa T, Ito J (2015). Insulin-like growth factor 1: A novel treatment for the protection or regeneration of cochlear hair cells. Hear Res.

[28] Wu J, Dong X, Li W, Zhao L, Zhou L, Sun S (2020). Dibenzazepine promotes cochlear supporting cell proliferation and hair cell regeneration in neonatal mice. Cell Prolif.

[29] Fang Q, Wei Y, Zhang Y, Cao W, Yan L, Kong M (2023). Stem cells as potential therapeutics for hearing loss. Front Neurosci.

[30] Xia M, Ma J, Sun S, Li W, Li H (2019). The biological strategies for hearing re-establishment based on the stem/progenitor cells. Neurosci Lett.

[31] Bonday ZQ, Cortez GS, Grogan MJ, Antonysamy S, Weichert K, Bocchinfuso WP (2018). LLY-283, a Potent and Selective Inhibitor of Arginine Methyltransferase 5, PRMT5, with Antitumor Activity. ACS Med Chem Lett.

[32] Zheng Z, Nan B, Liu C, Tang D, Li W, Zhao L (2023). Inhibition of histone methyltransferase PRMT5 attenuates cisplatin-induced hearing loss through the PI3K/Akt-mediated mitochondrial apoptotic pathway. J Pharm Anal.

[33] Zhao B, Zhang D, Sun Y, Lei M, Zeng P, Wang Y (2022). Explore the effect of LLY-283 on the ototoxicity of auditory cells caused by cisplatin: A bioinformatic analysis based on RNA-seq. J Clin Lab Anal.

[34] Zheng Z, Zeng S, Liu C, Li W, Zhao L, Cai C (2021). The DNA methylation inhibitor RG108 protects against noise-induced hearing loss. Cell Biol Toxicol.

[35] He Y, Zheng Z, Liu C, Li W, Zhao L, Nie G (2022). Inhibiting DNA methylation alleviates cisplatin-induced hearing loss by decreasing oxidative stress-induced mitochondria-dependent apoptosis via the LRP1-PI3K/AKT pathway. Acta Pharm Sin B.

[36] Zhang D, Sun Y, Lei M, Wang Y, Cai C (2023). Deciphering the potential ability of RG108 in cisplatin-induced HEI-OC1 ototoxicity: a research based on RNA-seq and molecular biology experiment. Hereditas.

[37] Fan K, Gao L, Yan X (2013). Human ferritin for tumor detection and therapy. Wiley Interdiscip Rev Nanomed Nanobiotechnol.

[38] Li L, Fang CJ, Ryan JC, Niemi EC, Lebrón JA, Björkman PJ (2010). Binding and uptake of H-ferritin are mediated by human transferrin receptor-1. Proc Natl Acad Sci U S A.

[39] Pozzi C, Di Pisa F, Bernacchioni C, Ciambellotti S, Turano P, Mangani S (2015). Iron binding to human heavy-chain ferritin. Acta Crystallogr D Biol Crystallogr.

[40] Theil EC (1987). Ferritin: structure, gene regulation, and cellular function in animals, plants, and microorganisms. Annu Rev Biochem.

[41] Clevers H (2006). Wnt/beta-catenin signaling in development and disease. Cell.

[42] Munnamalai V, Fekete DM (2013). Wnt signaling during cochlear development. Semin Cell Dev Biol.

[43] Shin S, Son Y, Liu KH, Kang W, Oh S (2019). Cytotoxic activity of broussochalcone a against colon and liver cancer cells by promoting destruction complex-independent β-catenin degradation. Food Chem Toxicol.

[44] Lustig B, Jerchow B, Sachs M, Weiler S, Pietsch T, Karsten U (2002). Negative feedback loop of Wnt signaling through upregulation of conductin/axin2 in colorectal and liver tumors. Mol Cell Biol.

[45] van Noort M, Meeldijk J, van der Zee R, Destree O, Clevers H (2002). Wnt signaling controls the phosphorylation status of beta-catenin. J Biol Chem.

[46] Jansson L, Ebeid M, Shen JW, Mokhtari TE, Quiruz LA, Ornitz DM (2019). β-Catenin is required for radial cell patterning and identity in the developing mouse cochlea. Proc Natl Acad Sci U S A.

