# Supplementary material for: An engineered ferritin nanocage co-delivery system for targeted hair cells protection and hearing loss attenuation
Source: Theranostics. 2026 Jul 13;16(14):8104–16. doi: 10.7150/thno.131600 (PMC13440433; doi:10.7150/thno.131600)
Supplement: Supplementary file 1 [file thnov16p8104s1.pdf]

Supplemental information

An engineered ferritin nanocage co-delivery system for targeted hair cells protection and hearing loss attenuation

Junying Zhang[1,2#], Zhiwei Zheng[1#], Jiaru Zhang[3#], Xinyuan Ma[4], Zihao Li[4], Hongyan Lin[4], Guohui Nie[5], Minmin Liang[2*], Hui Ding[5*], Yingzi He[1*]

[1] ENT Institute and Otorhinolaryngology Department of Eye & ENT Hospital, State Key Laboratory of Medical Neurobiology and MOE Frontiers Center for Brain Science, NHC Key Laboratory of Hearing Medicine (Fudan University), Shanghai, P. R. China.

[2] Experimental Center of Advanced Materials, School of Materials Science & Engineering, Beijing Institute of Technology, Beijing, P. R. China.

[3] Department of Ophthalmology, Beijing Chaoyang Hospital, Capital Medical University, Beijing, P. R. China.

[4] School of Basic Medical Sciences, Shanghai Medical College, Fudan University, Shanghai, P. R. China.

[5] Department of Otolaryngology and Institute of Translational Medicine, Shenzhen Second People's Hospital, the First Affiliated Hospital of Shenzhen University Health Science Center, Shenzhen, P. R. China.

[#] These authors made equal contributions.

[*]Correspondence: yingzihe09611@126.com (Y.H.); dinghui@email.szu.edu.cn (H.D.); mmliang@bit.edu.cn (M.L.)

Table S1 Physicochemical Properties of LLY283 and RG108

	Molecular weight	Chemical formula	Solubility in DMSO	Solubility in water	LogP
LLY283	342.35	$C_{17}H_{18}N_4O_4$	100 mg/mL	3.57 mg/mL	0.68
RG108	334.33	$C_{19}H_{14}N_2O_4$	100 mg/mL	< 0.1 mg/mL	1.96

Table S2. Primers for real-time PCR detection

Gene	Sequence 5′→3′
Mouse *Axin2*	F: TGACTCTCCTTCCAGATCCCA<br>R: TGCCCACACTAGGCTGACA
Mouse *β-catenin*	F: ATGGAGCCGGACAGAAAAGC<br>R: CTTGCCACTCAGGGAAGGA
Mouse *Tcf7l2*	F: AACGAACACAGCGAATGTTTCC<br>R: CTCGGCATTTCTTAGGAGCG
Mouse *β-actin*	F: GGCTGTATTCCCCTCCATCG<br>R: CCAGTTGGTAACAATGCCATGT

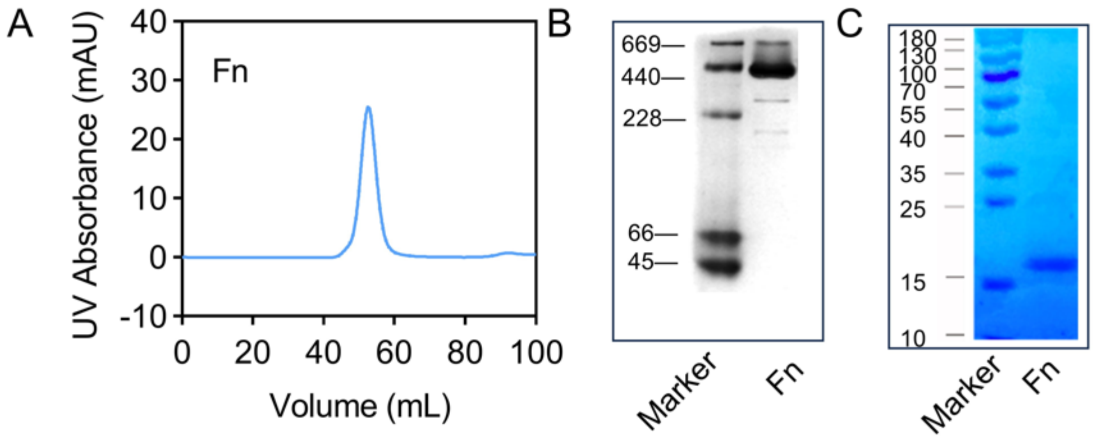

Figure S1 Characterization of purified ferritin. (A) Size-exclusion chromatography (SEC) profile of ferritin, showing a single symmetric peak corresponding to well-defined nanocage assembly. (B) Native polyacrylamide gel electrophoresis (Native-PAGE) analysis confirming the successful purification and structural integrity of ferritin. (C) SDS-PAGE analysis of ferritin after denaturation at 100 °C, followed by Coomassie Brilliant Blue staining, revealing a single protein band at approximately 21 kDa, corresponding to the ferritin heavy chain subunit. The experiments in (A), (B) and (C) were independently repeated three times with similar results.

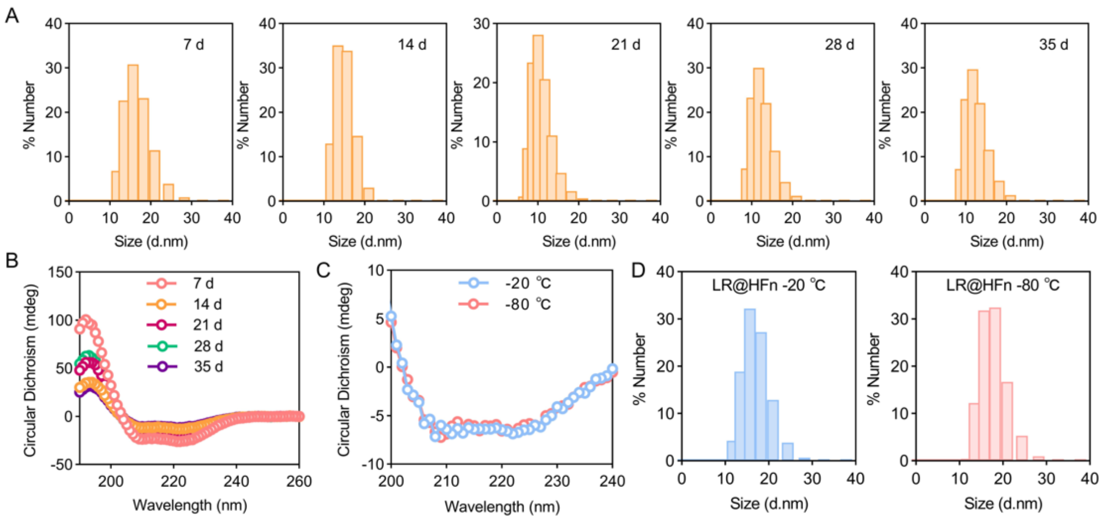

Figure S2 Stability evaluation of LR@Fn under different storage and processing conditions. (A) Particle size distribution of LR@Fn during storage, indicating its colloidal stability over time. (B) Circular dichroism (CD) spectrum of LR@Fn, confirming the preservation of its secondary structure after drug loading. (C) CD spectra of LR@Fn after freeze-drying and re-dissolution under different storage temperatures (−20 °C and −80 °C), demonstrating structural stability under cryogenic conditions. (D) Hydrodynamic particle size of LR@Fn after freeze-drying and re-solubilization at −20 °C and −80 °C, indicating minimal size variation and good re-dispersibility.

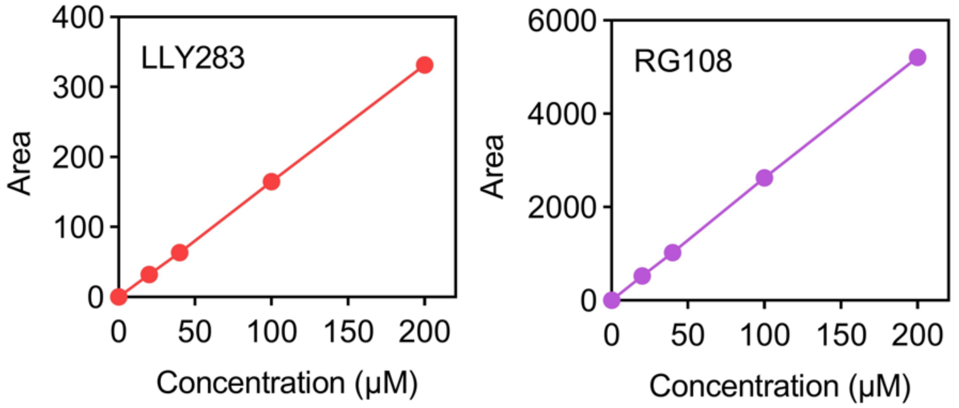

Figure S3. Calibration curves of LLY283 and RG108 established by high-performance liquid chromatography (HPLC). Standard curves were constructed using a series of known concentrations (0, 20, 50, 100, and 200 μM), demonstrating good linearity for quantitative analysis.

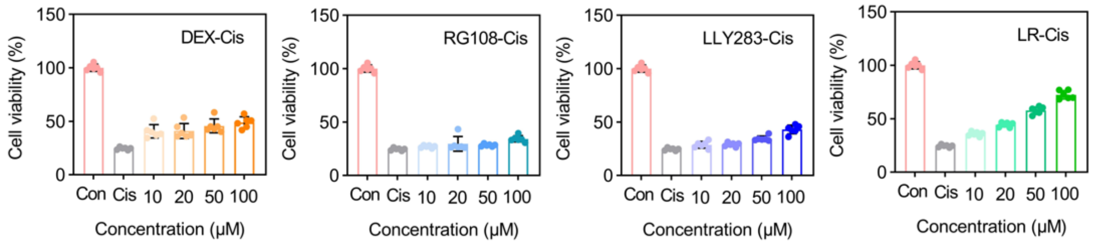

Figure S4. Cytotoxicity evaluation of small-molecule drugs in HEI-OC1 cells. Quantitative cell viability analysis of HEI-OC1 cells after treatment with DEX, RG108, LLY283, and LR at concentrations of 10, 20, 50, and 100 μM. Data are presented as mean ± SD (n = 6 independent experiments).

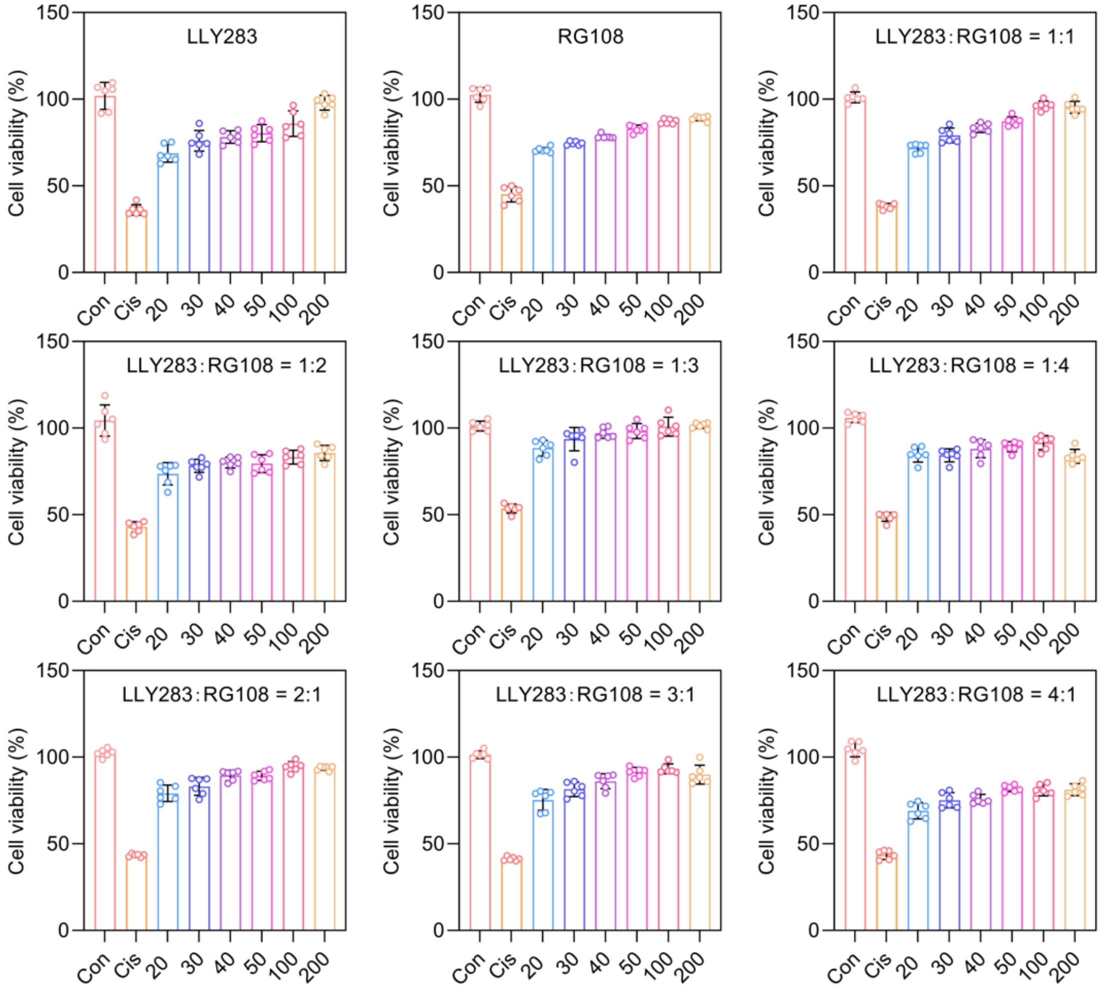

Figure S5. Dose- and ratio-dependent cytoprotective effects of LLY283 and RG108 evaluated by CCK-8 assay. Cell viability of HEI-OC1 cells following cisplatin-induced injury and treatment with LLY283, RG108, and their combinations at different ratios (1:4 to 4:1) under fixed total drug-equivalent concentrations (20–200 μM). All treatments exhibited dose-dependent protective effects. The cytoprotective efficacy showed a clear dependence on drug ratio, with the 1:3 (LLY283:RG108) ratio generally providing the most pronounced protection across multiple concentrations. Both

excessive RG108 (1:4) and excessive LLY283 (4:1) ratios resulted in reduced efficacy. In addition, the optimized combination demonstrated improved cytoprotective effects compared with either single-drug treatment at equivalent total concentrations. Data are presented as mean ± SD (n = 6).

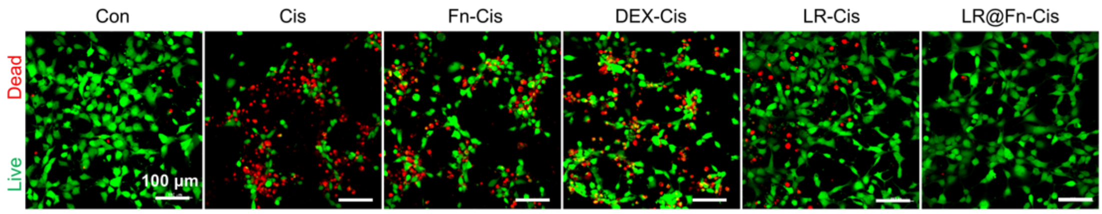

Figure S6. Live/dead staining analysis of HEI-OC1 cells under different treatments. HEI-OC1 cells were pretreated with PBS, Cis, DEX, RG108, LLY283, or LR@Fn for 2 h. Subsequently, Cis (20 μM) was added to the DEX, RG108, LLY283, and LR@Fn groups for an additional 20 h incubation. Cells were then subjected to live/dead staining to evaluate cell viability. All images were acquired under identical magnification. The experiments were independently repeated three times with similar results.

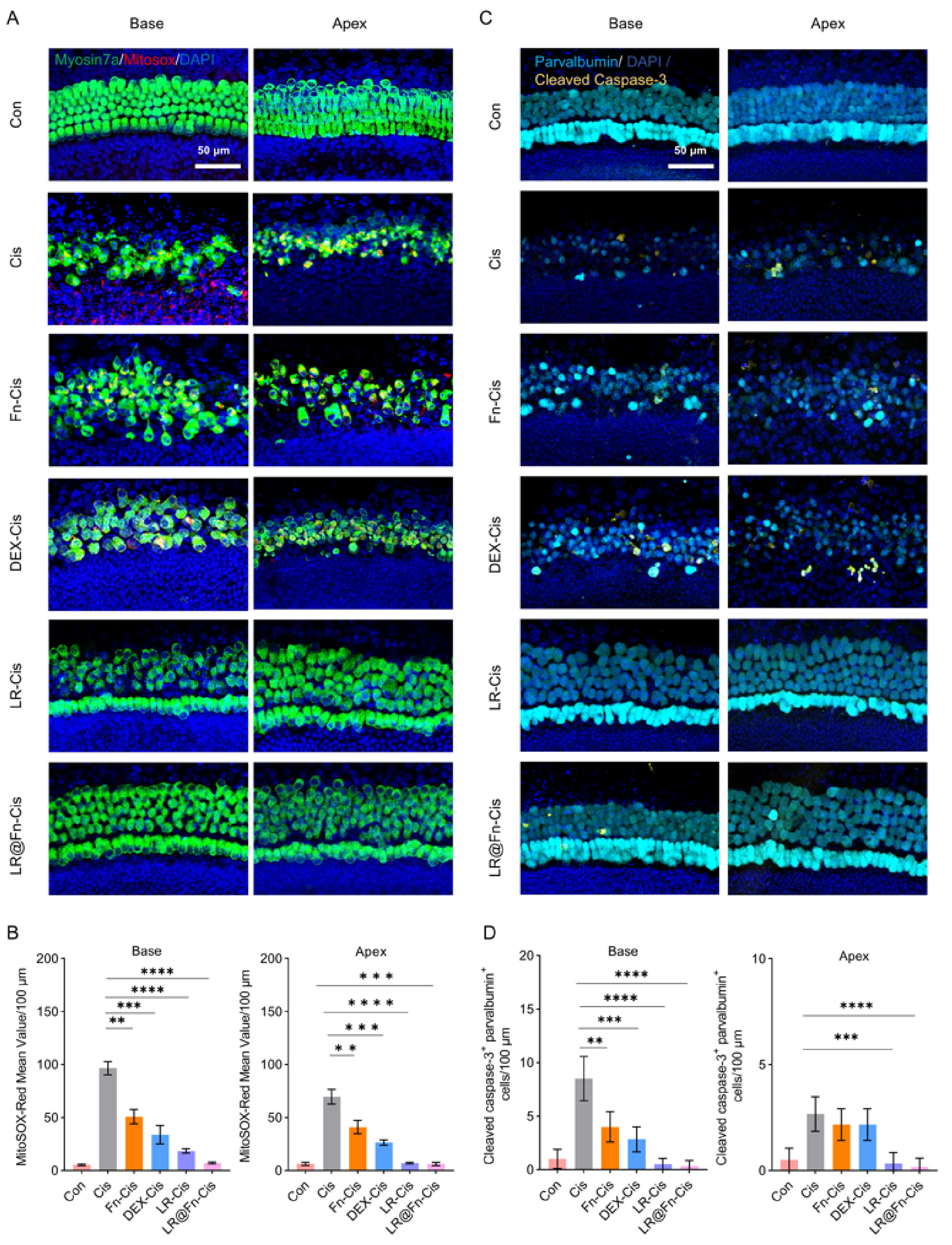

Figure S7 Effect of LR@Fn on cisplatin-induced basal and apical hair cell loss *ex vivo*. (A) Representative CLSM images of mitochondrial superoxide (red) in cochlear basal and apical cells after different treatments, with myosin 7a in green and DAPI in blue. The images are presented at the same magnification. (B) Quantification of cochlear basal and apical cell mitochondrial superoxide positive fluorescence. (n = 3). After different treatments, a region was selected for counting every 100 μm along the middle region of the cochlea. (C) Representative CLSM images of apoptotic markers in cochlear basal and apical cells after different treatments (yellow), hair cells and DAPI in light blue and dark blue. These images are presented at the same magnification. (D)

Positive counts of apoptotic markers in cochlear basal and apical cells (n = 6). After different treatments, a region was selected for counting every 100 μm along the middle region of the cochlea. The data in (B) and (D) are presented as the mean ± S.D. and were compared via one-way ANOVA. The experiments in (A) and (C) were repeated independently three times with similar results. ns. not significant, *p<0.05, **p<0.01, ***p<0.001, ****p<0.0001.

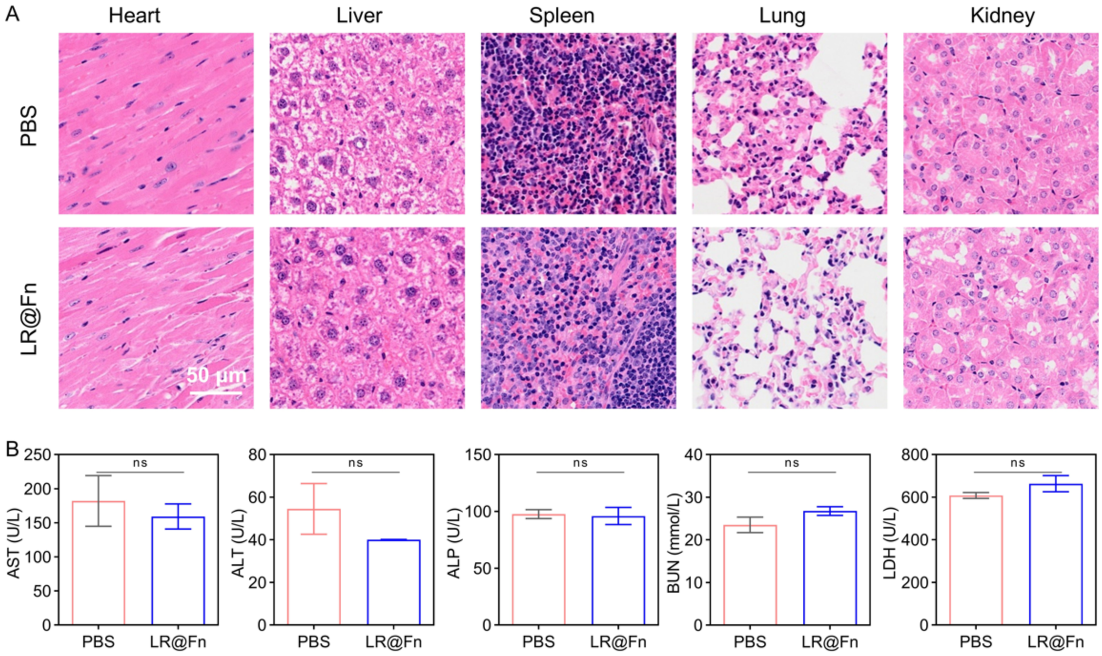

Figure S8 The safety evaluation of LR@Fn. (A) HE staining analysis. Major organs including the heart, liver, spleen, lung, and kidney of C57BL/6J mouse in PBS and LR@Fn groups were taken, and the analysis showed that LR@Fn showed no observable toxicity. (B) Blood biochemical index analysis. The blood of PBS and LR@Fn group after injection was taken, and the analysis showed that all parameters remained within normal physiological ranges and no toxicity was found. The data in (B) are presented as the mean ± S.D. and were compared via two-tailed unpaired Student's t-test. The experiments in (A) were repeated independently three times with similar results. ns. not significant.

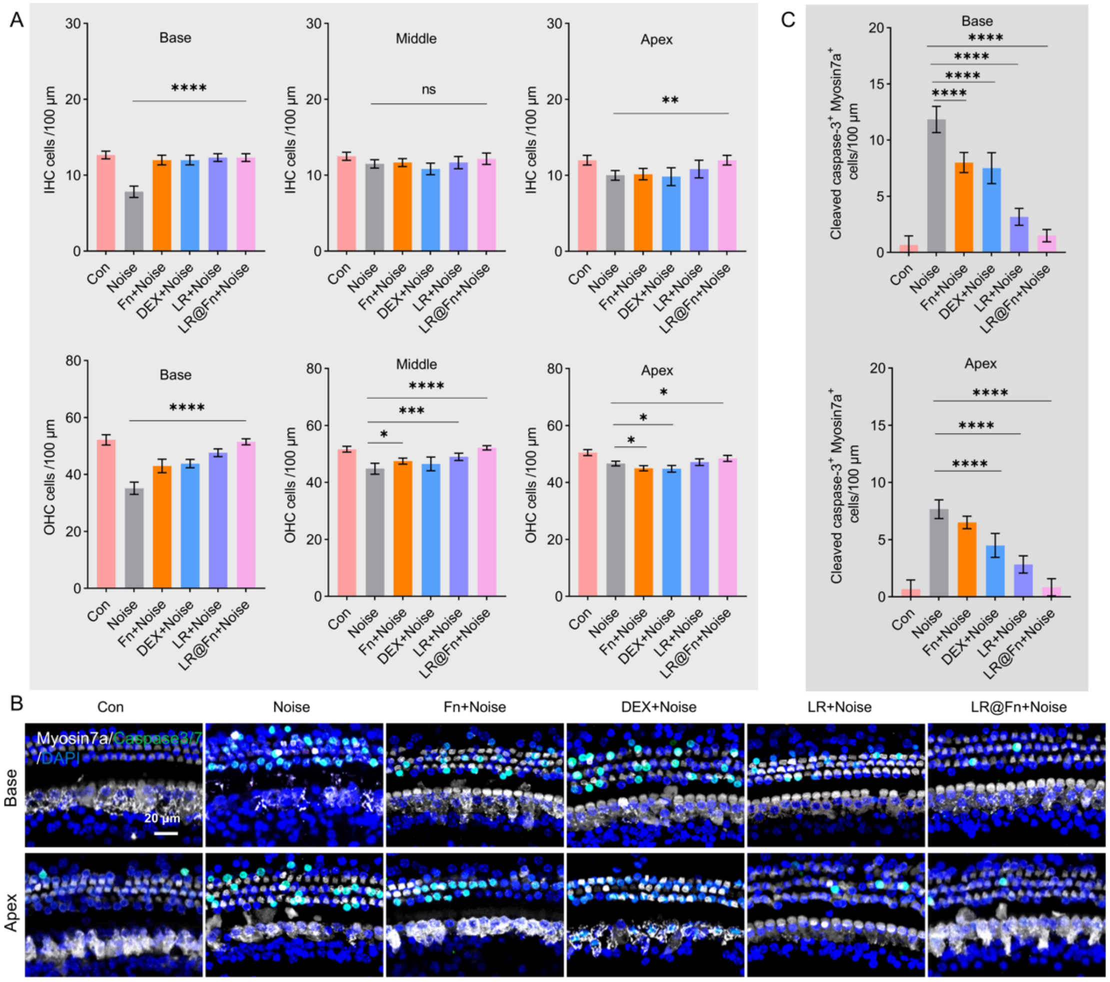

Figure S9 Effect of LR@Fn on noise-induced hearing loss *in vivo*. (A) Quantitative analysis of the number of inner hair cells and outer hair cells corresponding to the basal, middle and apical regions of the cochlea in different treatment groups (Noise, Fn + Noise, DEX + Noise, LR + Noise, LR@Fn + Noise). (n=6, 1 cochlea from each of 6 mice). (B) Representative CLSM images of cochlear basal and apical hair cells apoptotic markers (green) after different treatments, white for myosin 7a (white) and blue for DAPI. The images are presented at the same magnification. (C) Counts of cochlear basal and apical hair cells positive for apoptotic markers (n = 6, 1 cochlea from each of 6 mice). The data in (A) and (C) are presented as the mean ± S.D. and were compared via one-way ANOVA. The experiments in (B) were repeated independently three times with similar results. ns, not significant, *p<0.05, ***p<0.001, ****p<0.0001.
